# Virulence-Related Genes Identified from the Genome Sequence of the Non-O1/Non-O139 Vibrio cholerae Strain VcN1, Isolated from Dhaka, Bangladesh

**DOI:** 10.1128/genomeA.01513-17

**Published:** 2018-03-08

**Authors:** Maqsud Hossain, Munirul Alam, Abdul Khaleque, Sohidul Islam, Abdus Sadique, Nayeim Khan, Zahra Halim, Mrinmoy Sarker, Najib M. El-Sayed, Anwar Huq, Gias Uddin Ahsan, Rita R. Colwell

**Affiliations:** a Department of Biochemistry and Microbiology, North South University, Dhaka, Bangladesh; b NSU Genome Research Institute, North South University, Dhaka, Bangladesh; c International Centre for Diarrheal Disease Research, Bangladesh (ICDDR,B), Dhaka, Bangladesh; d Maryland Pathogen Research Institute, University of Maryland, College Park, Maryland, USA; e Center for Bioinformatics and Computational Biology, University of Maryland, College Park, Maryland, USA; f Department of Public Health, North South University, Dhaka, Bangladesh; g University of Maryland Institute of Advanced Computer Studies, University of Maryland, College Park, Maryland, USA; h Johns Hopkins Bloomberg School of Public Health, Baltimore, Maryland, USA

## Abstract

We report here the first draft genome sequence of the non-O1/non-O139 Vibrio cholerae strain VcN1, isolated from Dhaka, Bangladesh. The data submitted to GenBank for this strain will contribute to advancing our understanding of this environmentally disseminated bacterium, including its virulence and its evolution as an important pathogen.

## GENOME ANNOUNCEMENT

Vibrio cholerae, the causative agent of cholera, is a Gram-negative bacterium that is adapted to the aquatic environment and is also a human pathogen. Of more than 200 known serogroups of V. cholerae, O1 and O139 are associated with a major virulence factor cholera toxin (CTX) and with epidemic cholera worldwide (1). The noncholera, or non-O1/non-O139, serogroups rarely carry the CTX gene cassette, but they can serve as a reservoir for virulence and related genes routinely found in cholera serogroup strains (2) and can cause diarrhea and extraintestinal infections (3).

Here, we report the draft genome sequence of the non-O1/non-O139 V. cholerae strain VcN1, isolated from a river in Dhaka, Bangladesh. A genomic library was constructed and employed for 300-bp paired-end whole-genome sequencing using an Illumina MiSeq platform (Illumina, San Diego, CA, USA) at the Genome Research Institute of North South University, Bangladesh. A total number of 293,954 raw reads were generated (∼15× coverage) and assembled using SPAdes version 3.11 (4). The scaffold generated was mapped and ordered using ABACAS (5) and included the reference genome of V. cholerae O1 El Tor strain MS6 (GenBank accession no. AP014524 for chromosome 1 and AP014525 for chromosome 2). Structural gene prediction and functional annotation were performed using the Rapid Annotations using Subsystems Technology (RAST) server (6).

The total size of the draft assembly was 4,146,313 bp, arranged into 178 contigs with an *N*_50_ of 89 kb. The GC content was determined to be 47.57%. After scaffolding using the reference genome, 40 contigs totaling 2,997,625 bp in size mapped to chromosome 1 and 33 contigs totaling 1,113,511 bp in size mapped to chromosome 2. Another 105 contigs, with a cumulative size of 906,989 bp (*N*_50_ = 80 kb), did not map to the reference genome.

Several genes, gene clusters, and operons responsible for virulence, disease, and defense mechanisms were detected. Although the cholera enterotoxin *ctx* was absent, other known virulence factors, such as *zot* (zonula occludens toxin) and *toxr* and *toxs* (regulators of the expression of cholera pathogenicity) (7), were detected in the genome. Genes coding for resistance to antibiotics and toxic substances were also detected, including those for fluoroquinolone and tetracycline resistance, multidrug resistance efflux pumps, and the multidrug resistance tripartite system found in Gram-negative bacteria.

In future studies, the whole-genome sequence of V. cholerae VcN1 and additional strains of other cholera serogroups of V. cholerae will be analyzed using comparative genomics to understand differences in virulence and related factors, the incidence of antibiotic resistance genes, genome plasticity, and the evolutionary dynamics of this important human pathogen.

### Accession number(s).

This whole-genome shotgun project has been deposited in GenBank under the accession no. PDNJ00000000.
